# Linkage and haplotype analysis for chemokine receptors clustered on chromosome 3p21.3 and transmitted in family pedigrees with asthma and atopy

**DOI:** 10.4103/0256-4947.60516

**Published:** 2010

**Authors:** Saleh A. Al-Abdulhadi, Mohammed W. O. Al-Rabia

**Affiliations:** a From the Department of Child Health, Royal Aberdeen Children's Hospital, University of Aberdeen, Medical School, Westburn Road, Aberdeen, Scotland, United Kingdom; b From the Department of Hematology and Immunology, College of Medicine, Umm Al-Qura University, Makkah, Saudi Arabia

## Abstract

**BACKGROUND AND OBJECTIVES:**

Genomic scan analyses have suggested that the chemokine receptor cluster (CCR2, CCR3, CCR5 <300 kb span) on the short arm of chromosome 3 may contribute to susceptibility to HIV-1 infection and to the expression of a number of inflammatory diseases. Two single nucleotide polymorphisms (SNP) and a deletion in these chemokine receptors have also been found in case-control studies to be associated with susceptibility for asthma and related phenotypes. We extended these case-control studies by establishing whether these polymorphisms were in linkage and linkage disequilibrium with asthma and related phenotypes using linkage and haplotype analyses.

**METHODS:**

We genotyped 154 nuclear families identified through two child probands with physician-diagnosed asthma (453 unrelated individuals) including 303 unrelated parents and 150 unrelated children. Atopy was defined as a positive skin prick test (SPT 3 mm) to a panel of common inhaled allergens.

**RESULTS:**

From a panel of ten known SNPs, only three polymorphisms: –G190A in CCR2, –T51C in CCR3, and a 32 bp deletion in CCR5 were found to occur at clinically relevant frequencies. All 154 families were used for haplotype analysis but only 12 nuclear families were eligible for linkage analysis. Both analyses confirmed that the mutations were in linkage with asthma, but not with atopy.

**CONCLUSION:**

The chemokine receptor genes on 3p21.3 are significantly plausible candidate genes that can influence the expression of asthma. The previous association of the CCR5Δ32 deletion with protection from childhood asthma appears to be explained by linkage disequilibrium with the –G190A mutation in the CCR2 receptor gene.

Asthma is a complex disease which in childhood is frequently, but not invariably, associated with atopy,^1^ and which is only associated with approximately half of all adult cases.^2^ Although is likely that many candidate genes contribute to disease expression in view of asthma's high population prevalence, candidate polymorphisms are also likely to have high population frequencies and play relevant roles in biologically plausible mechanistic pathways. The chemokine pathway is among the many plausible pathways involved in the underlying inflammatory airway responses that are typical of asthma.

Chemokines, or chemotactic cytokines, are secretory proteins produced by leucocytes and tissue cells, either constitutively or after induction.^3^ The two main subfamilies, CXC and CC chemokines (also termed α and β chemokines), are distinguished according to whether the position of the first two cysteines are separated by one amino acid (CXC) or are adjacent to each other (CC). Cysteines form two disulphide bonds (cys1→cys3 and cys2→cys4), which confer to the chemokines their characteristic three-dimensional folding.^3^ Receptors for chemokines belong to the seven transmembrane-spanning receptor family and the majority are G protein-coupled receptors.

Chemokines have been implicated in both inflammatory and homeostatic leukocyte migrations.^4^ In contrast, CC chemokines generally attract monocytes and lymphocytes but not neutrophils. Several CC members have been shown to induce eosinophil migration, including eotaxin-1, eotaxin-2, MCP-2, MCP-3, MCP-4, RANTES, MDC, and MIP-1α, and many of these same chemokines also act on basophils. The selectivity of the CC subfamily for T cells, monocytes, eosinophils, and basophils has led to numerous studies of their roles in allergic inflammation. Several members of the C, CC, and CXC chemokine subfamilies induce T-cell migration. Polarization of T-cells into TH1 and TH2 is characterized by differences in chemokine receptor expression. For example, subsets of TH2 cells have been shown to selectively express CCR3, which plays a role in basophil and eosinophil recruitment, reinforcing the suggestion that CCR3 is an important chemokine receptor in the expression of allergic disease. CCR1 and CCR5 have also been reported to be markers of TH1 cells and there is circumstantial evidence that TH1 and TH2 cells may recruit additional, polarized T cells by amplifying loops involving selective chemokine induction by IFN-γ and IL-4.^5^

It has been suggested that there may be some contribution of the functional mutations in the chemokine pathway in several diseases including rheumatoid arthritis^6^, HIV-1 infection,^7^,^8^ atopic dermatitis,^9^ and childhood asthma.^10^ Activation of different chemokine receptors in the same cell type may produce distinct signals, one example being CCR2 engagement by monocyte chemoattractant protein (MCP-1) and histamine release in basophils.^11^ Particular interest has been directed to the role of receptor internalization in the infection of susceptible cells by HIV, a process that can be mediated by CCR5, CXCR4, or other chemokine receptors in a virus strain-dependent manner. Generation of chemokine receptor knockout animals has been instructive as mice deficient in MCP-1 or its receptor, CCR2, have impaired host defence, hematopoiesis, TH1/TH2 balance, monocyte macrophage recruitment, and an increased formation of atherosclerotic lesions.^12^ The CCR3 receptor is important in the movement of eosinophils, basophils, and possibly TH2 cells. Chemokine binding initiates rapid internalization but no signal transduction.

Most of the genes encoding CC chemokine receptors including CCR1 to CCR9 have been mapped to chromosome 3p21.3-p24, and of these, CCR2, 3, and 5 are clustered within a total distance of 350 kb on chromosome 3p21.3.^13^ The coding region of the majority of chemokine receptor genes resides on one single exon, with the exception of CCR7, which contains two introns interrupting the coding region of the domain.

In view of the complex interdependencies, redundancies, and in particular, the close genomic distance between three of the strongest and most studied candidates in human disease expression, we assessed the linkage and the linkage disequilibrium between SNPs in CCR5, CCR2, and CCR3 in families at high risk for asthma and atopy.

We analyzed numbers of SNPs within the chemokine receptors CCR2, CCR3, and CCR5 using DNA pooling and allelic quantification (AQ) to identify two most common SNPs in our population. Three highly allelic single nucleotide polymorphisms were observed: –G/A substitution at position 190 of the CCR2 gene changes the amino acid at position 64 from isoleucine to valine of its protein, –T/C substitution at position 51 of the CCR3 gene results in a silent mutation at position 17 of its protein, and a 32 base pair deletion within the CCR5 gene clustered in chromosome 3p21.4 within a 300 kbp span were examined by the linkage disequilibrium and linkage analysis for asthma and atopy.

## METHODS

### Subjects

Population characteristics have been described in detail elsewhere.^14^,^15^ Briefly, we identified 154 unrelated nuclear families (598 individuals including children and parents) containing at least two children and young adults aged 8-24 yrs, the majority of whom had a physician's diagnosis of asthma (PDA) and current symptoms (wheezing in the past 12 months). Lung function was measured by spirometry according to American Thoracic Society (ATS) standards (ATS; 1991). Bronchial hyperresponsiveness BHR was assessed with a methacholine challenge test and the concentration (mg/mL) was noted at which there was a fall in FEV_1_ of 20% (PC_20_).^16^ All 154 families were white Caucasian and were drawn from a population for which the UK 2001 census showed that 84% were born in Scotland, 12% in other parts of the UK, and only 4% outside the UK.

Atopy was defined as the presence of at least one positive skin prick test (SPT) with a wheal size of ≥3 mm according to the European guidelines^17^ for five inhalant allergens (cat, dog, house dust mite, grass, and *Alternaria*) referenced to a negative control. These five allergens were selected according to our own clinical survey and patient questionnaire for atopic patients. This survey confirms that these five allergens are the most common in our populations. DNA was isolated from EDTA-treated whole blood using the phenol-chloroform method.

Consent was obtained from each participant and approval was awarded for all the studies by the Grampian Research Ethics Committee, our local IRB.

### DNA pooling and allelic quantification

This protocol has been described in detail elsewhere.^14^,^15^ Briefly, each prePCR pool was amplified by PCR for all candidate SNPs for each gene in triplicate using the following PCR conditions: 50 μL containing 100 ng of genomic DNA; 10X PCR Buffer; 25 mM MgCl_2_; 10 mM dNTPs; 10 pmol/μL of each primer (Table 1). For each run, 5 U of *Taq* DNA polymerase was used in 40 cycles of 95°C for 45 seconds, 60°C for 45 seconds, and 72°C for 1.5 minutes.^14^,^15^ The PCR products (postPCR pools) were inspected for clearly scored products using electrophoresis (1.5% agarose gels stained with ethidium bromide). Thirty microliters of each postPCR pool were then used to determine allelic frequencies using Pyrosequencing™ technology (PSQ).^18^ The PCR products were prepared for PSQ using a PSQ96 Sample Prep tool and PSQ reactions were performed using the PSQt96 SNP reagent kit according to the manufacturer's instructions with two different sequencing primers for each SNP (Table 1); each run was performed in triplicate. Each PSQ96 plate contained one negative control (no template) and one positive control per genotype. The program, PSQt96 evaluation allelic quantification (AQ) software, was used to obtain the ratio of each separate allele peak height to the sum of the heights of both allele peaks. To allow the conversion of this peak height ratio to allele frequency for the DNA pools, a standard curve was established based on individual samples and the ratios from the pooled samples plotted against individual sample frequency. The equation of the linear regression best-fit line was then determined for each SNP and used to convert the allele peak height ratios to allele frequencies in the DNA pools. All eight known SNPs in genes encoding for CCR2 and CCR3 were assessed to identify those with a clinically significant population frequency in our population. CCR5 SNPs were not assessed as we had previously established the possible relevance of the CCR532 polymorphism in our population.^10^

**Table 1:** Candidate SNPs and DNA pooling and sequencing oligonucleotide primers.

Chemokines	Assay	Descriptions	5'-Sequence-3'
CCR2	SNP1,2,3	PCR-Forward primer	Biotin-CGGTGCTCCCTGTCATAAAT
		PCR-Reverse primer	AGCCCACAATGGGAGAGTAA
	SNP1	V64I-PSQ-Sequencing primer	TTTTGCAGTTTATTAAGATGA
	SNP2	V52V-PSQ-Sequencing primer	CCCACAAAACCAAAGATG
	SNP3	L45V-PSQ-Sequencing primer	AGTAGAGCGGAGGCA
	SNP 4	PCR-Forward primer	Biotin-GAGGCATAGGGCAGTGAGAG
		PCR-Reverse primer	CTGAACTTCTCCCCAACGAA
	SNP4	N260N-PSQ-Sequencing primer	TGTTCAGGAGAATGACAA

CCR3	SNP 1,2	PCR-Forward primer	Biotin-CACATGTGGCATCTTTGTTG
		PCR-Reverse primer	GGCCATCAGTGCTCTGGTAT
	SNP1	G21D-PSQ-Sequencing primer	CTTTTTCACAGAGCAGG
	SNP2	5'UTR-PSQ-Sequencing primer	TGAGGTTGTCATTTCACTT
	SNP 3	PCR-Forward primer	CACATGTGGCATCTTTGTTG
		PCR-Reverse primer	Biotin-GGCCATCAGTGCTCTGGTAT
	SNP3	Y17Y-PSQ-Sequencing primer	CCTTTGGTACCACATCCT
	SNP 4	PCR-Forward primer	CCTGCTCTGTGAAAAAGCTGA
		PCR-Reverse primer	Biotin-GATCATCACCACCACCACAT
	SNP4	T39C-PSQ-Sequencing primer	CCAGTTTGTGCCCC

CCR2	V64I	TaqMan-Forward primer	CGGTGCTCCCTGTCATAAATTTGA
		TaqMan-Reverse primer	GTCAGTCAAGCACTTCAGCTTTT
		TaqMan-Probe 1	VIC-ACATGCTGGTCATCCT-NFQ
		TaqMan-Probe 2	FAM-ACATGCTGGTCGTCCT-NFQ

CCR3	Y17Y	TaqMan-Forward primer	AGTTGAGACCTTTGGTACCACATC
		TaqMan-Reverse primer	ATCAGTGCTCTGGTATCAGCTTTT
		TaqMan-Probe 1	VIC-CCACGTCATCGTAGTAG-NFQ
		TaqMan-Probe 2	FAM-CCACGTCATCATAGTAG-NFQ

CCR5	Δ32	PCR-Forward primer	TGTTTGCGTCTCTCCCAG
		PCR-Reverse primer	CACAGCCCTGTGCCTCTT

### Genotyping and sequencing

Those SNPs with possibly relevant allele frequencies (above 2%) (Table 2) were genotyped and sequenced in each individual. Primers and probes were designed by Assay-by-design (Applied Biosystem) and probe lengths were adjusted such that both probes had approximately the same melting temperature (67°C). The probe melting temperature was 7.0-8.0°C above the primer melting temperature of 60°C. Out of the two fluorogenic allele-specific probes used, one matched the wild-type sequence and other matched the mutant sequence. Each probe was labeled at the 5′ end with a fluorescent reporter dye and at the 3′ end with the quencher dye, NFQ. The reporter dyes used were VIC for the wild-type sequence and FAM for the mutant sequence. TaqMan universal master mix (Applied Biosystem) was used at 1X final concentration and in a volume of 25 uL along with 50 ng of genomic DNA and sequencing primers (Table 1). Optical plates (Applied Biosystem) were thermocycled in the Prism 7700 Sequence Detection System (SDS) for real-time detection and end-point analysis.

**Table 2:** Candidate SNPs, position, function and observed allelic frequencies.

Chemokines	SNPs	Location	AA change	Function	Observed cases (%)	Observed controls (%)	NCBI (%)
CCR2	C/G	Coding Exon 3	V45L	Nonsynonymous	0.20	0.31	0.5
CCR2	G/T	Coding Exon 3	V52V	Synonymous	0.16	0.22	0.4
CCR2	A/G	Coding Exon 3	I64V	Nonsynonymous	6.3	21.0	15.2
CCR2	T/C	Coding Exon 3	N260N	Synonymous	0.7	0.63	1.41
CCR3	C/T	Coding Exon 4	Y17Y	Synonymous	14.2	3.5	16.9
CCR3	A/G	Coding Exon 4	D21G	Nonsynonymous	0.2	1.6	1.3
CCR3	T/C	Coding Exon 4	L39P	Nonsynonymous	0.1	0.5	0.4
CCR3	A/G	5'UTR	Unknown	Unknown	0.2	0.3	0.2

NCBI: National Centre of Biotechnology Information.

The CCR5Δ32 polymorphism was genotyped using a PCR assay as follows: PCR was performed in 25 μL containing 100 ng of genomic DNA; 10X PCR Buffer; 25 mM MgCl_2_; 10 mM dNTPs; 10 pmol/μL of each primer (Table 1), and 5 U of Taq DNA polymerase. PCR conditions were 40 cycles of 95°C for 45 seconds, 60°C for 45 seconds, and 72°C for 1.5 minutes. The PCR products, 232 bp for wild-type allele and 200 bp for the deletion Δ32 allele, were resolved by electrophoresis on 1.5% agarose gels stained with ethidium bromide.

### Statistical analyses

The LINKAGE package used to assess the likelihood of the recombination fraction between loci of interest expressed as the common logarithm of the ratio or lodscore which was calculated using the MSIM program. Multipoint linkage analysis was performed using the MLINK, LINKMAP, VITESSE, and LINKLODS computer packages.^19^,^20^

The most common application of linkage analysis is to identify the location of a gene responsible for a particular Mendelian inherited disease. We sought to estimate the linkage between three genetic loci in chromosome 3p21.3 and asthma.

Only 12 out of 154 families had heterozygous parents for all three polymorphisms within the CCR5, CCR2, and CCR3 genes, and were therefore eligible for linkage analyses.

### Haplotype association

Haplotype analyses were constructed from population genotype data and using the Haplotyper program.^21^ Linkage disequilibrium between each polymorphism was estimated by using the Fisher exact test and the 2BY2 program and the output generated by the EH (Estimate Haplotype) program.^22^ Bonferroni adjustment on the number of independent comparisons^23^ was made on the correlation coefficients for each of the haplotypes tested to correct for the number of comparisons made. The same approach was used to correct for the number of polymorphisms tested by using the D values instead of the correlation coefficients to estimate the relatedness of the polymorphisms.

## RESULTS

Simulation of linkage was performed to check the power of this study to detect linkage with asthma status as the phenotype. Genotyping was simulated using the SLINK program^24^,^25^ for polymorphic SNPs that were located at three-recombination distances (θ) from each other. The three values for theta were therefore 0.001, representing a 1% recombination rate; 0.05, representing a 5% recombination rate; and 0.50, representing a 50% recombination rate (no linkage between the any of the three hypothesized SNPs). The polymorphic SNPs had two-allele frequencies for each investigated gene and corresponded to heterozygosity of 35.3, 26.8, and 16.9% for the CCR5, CCR2, CCR3 polymorphisms respectively (data not shown). The hypothetical trait allele was assigned a frequency of 0.261, 0.195, and 0.135 for CCR5, CCR2, and CCR3 respectively. Expected and maximal lodscores at different values of recombination fraction as well as the probability that the maximal lodscore will exceed a given value were calculated from 200 replicates using the analysis program MSIM^24^,^25^ (data not shown). The average lodscores are indicative of the information content of the pedigree studied for linkage analysis, whereas the probability that the maximal lodscore exceeds a given constant is a direct estimate of the power of the analysis. The simulation analysis showed that the pedigrees used in this study were capable of giving average and maximal lodscores of 1.57 and 6.08 respectively, with an estimated θ=0.05 between all four proposed disease loci. The study also showed that there was 89.5%, 44.3%, and 10.0% probability of obtaining lodscores >1.0, >2.0, and >3.0 respectively, at true θ=0.01, provided that the initial assumptions were correct. The probability of obtaining a false positive result, represented by the percentage of maximal lodscore values >3.0 when θ=0.50 (no linkage), was found to be 0.0% in this study.

To determine the inheritance pattern at chromosome 3p21.3, we genotyped 453 individuals in multiplex families for three genetic markers. Two-point linkage analysis was performed using the program MLINK^26^ which calculates two-point lodscores at different predefined recombination fractions (θ). The θ values were defined from 0.05 to 0.5 at an increment of 0.05. Multipoint linkage analysis was then performed to detect linkage to asthma using the LINKMAP, VITESSE, and LINKLODS computer packages (Table 3).^19^,^20^ LINKMAP and VITESSE generate maximum log likelihoods as they move the proposed disease locus across a fixed gene map. Recombination fractions and gene order for the three genes used in this study were derived from the National Centre for Biotechnology Information (NCBI) database. Maximal log likelihoods were converted to multipoint lodscores using the program LINKLODS. The highest multipoint lodscore of 3.9 was obtained in pedigree 8 at CCR5Δ32 (θ=0.00) and 0.22 at CCR3Y17Y (θ=0.00) in all pedigrees.

**Table 3:** Two-point lodscore at θ=0.00 from the CCR5Δ32, CCR2 V64I, and CCR3 Y17Y polymorphisms.

Pedigree	Two-point lodscore at θ=0.00		
	CCR5Δ32	CCR2I64V	CCR3Y17Y
1	1.26	−4.19	0.22
2	3.86	0.06	0.22
3	2.43	0.00	0.22
4	1.30	0.16	0.22
5	3.01	0.00	0.22
6	3.00	0.00	0.22
7	3.29	0.25	0.22
8	3.90	0.00	0.22
9	3.18	0.00	0.22
10	3.01	−0.39	0.22
11	3.89	0.00	0.22
12	3.74	−0.22	0.22

### Haplotype associations

If the population contains only a limited number of disease-predisposing ancestral alleles, affected individuals should show an excess of those ancestral haplotypes. To search for such association, we examined each chromosome in multiplex families and determined the haplotypes for three genetic markers. Depending on the mode of action of the putative predisposing genes, the trait-associated chromosomes will also contain normal chromosomes but, nonetheless, should still be enriched for disease-predisposing alleles if chromosome 3p21.3 plays a role in that trait.

An EH algorithm computer program was used to enumerate all possible haplotypes of adjacent markers by considering all haplotype lengths. For each haplotype, we counted the frequency of its occurrence among trait-associated and controls. Visual inspection suggested a clustering of apparently identical segments between distinct haplotypes in the region of these chemokine receptors, possibly reminiscent of a conserved founder haplotype. Analysis was done of the relationship between the genotypes defined by the combination of CCR5Δ32, CCR2-G190A, and CCR3-T51C polymorphisms in relation to asthma with or without atopy. The frequencies of individuals and haplotypes meeting for unrelated parents and children, cases, and controls are shown in Table 4. The frequencies of haplotype analyses by EH algorithm for case-control parents and children displayed in Table 4 suggested a linkage disequilibrium between these polymorphisms (χ^2^=14.62, *P*=.05). The Fisher exact test statistical analysis was applied to calculate Haplotype association and asthma status. Cross-tabulation statistical analyses were applied to calculate haplotype association and asthma status. These analyses suggested that haplotype 2 was associated with a high risk of asthma (*P*=.01), and haplotypes 3 and 4 were associated with controls (*P*=.0001).

**Table 4:** Haplotype analyses for parents and children.

Haplotype	SNPs			Parents (N=303)		Children (N=150)	
	Δ32	164V	Y17Y	Cases (%)	Controls (%)	Cases (%)	Controls (%)
1	1	1	1	58 (74)	164 (71)	61 (64)	38 (68)
2	1	1	2	6 (8)	9 (4)	14 (15)	4 (7)*
3	1	2	1	3 (4)	29 (13)**	5 (5)	5 (10)
4	1	2	2	0 (0)	2 (0.6)**	0 (0)	0 (0)
5	2	1	1	10 (13)	22 (10)	11 (12)	7 (13)
6	2	1	2	1 (1)	1 (0)	1 (1)	1 (2)
7	2	2	1	0 (0)	4 (2)	3 (3)	0 (0)
8	2	2	2	0 (0)	0 (0)	0 (0)	0 (0)

**Total**				**78**	**231**	**95**	**55**

* *P*<.01

** *P*<.001 (Chi-χ^2^)

## DISCUSSION

Genetic linkage analyses have been successful in the identification of genes involved in many single gene disorders such as cystic fibrosis and have also been used with limited success in disorders involving many genes and environmental factors such as hypertension^27^, colon cancer^28^, and diabetes mellitus.^29^

In the current study, an attempt was made to use genetic linkage analysis to assess the role of the chromosome 3p21.3 region in asthma with or without atopy. Computer simulation revealed the limited power of the pedigrees used in this study by showing that there was only 10% probability of obtaining a lodscore >3.0 for a marker located at θ=0.01 from the hypothetical trait locus. Actual linkage analysis performed on the 12 pedigrees showed positive values for lodscores across the entire region of chromosome 3p21.3 with a maximum value of 3.90 at the CCR5 gene (θ=.00). High LOD scores for the Δ32 allele, compared to the theoretical maximum, might have been in part due to the layout of the parameter files and the fact that the Δ32 allele had a high allele frequency in the present population. Although a dominant effect was assumed in the analysis, other models, including recessive or partial penetrance, would have been unlikely to change the overall results because of the high allele frequency and the large number of affected individuals in the families studied. This might limit the detection of linkage, especially for a complex disease such as asthma.

Haplotype analyses with the EH program suggested significant linkage disequilibrium between hypothetical SNPs within chemokine receptors and asthma and supported the suggestion that the -G190A polymorphism of CCR2 was protective against asthma.^13^,^14^ The relationship was analyzed between genotype defined by the combination of the CCR5Δ32, CCR2V64I, and CCR3Y17Y polymorphisms and asthma with or without atopy. The frequencies of individuals and haplotype meeting for unrelated parents and children cases and controls suggested linkage disequilibrium between these polymorphisms. The analysis also suggested that it was the CCR2-G190A polymorphism rather than the CCR5Δ32 that was associated with protection against asthma as this protective association was independent of its co-association with CCR532. The -T51C polymorphism in CCR3 was in significant linkage disequilibrium with the wild types of CCR5 and CCR2, but had an association with increased asthma risk, independent of its tendency to be in linkage disequilibrium with the above two wild type polymorphisms.

In summary, the present study demonstrated the utility of linkage analysis in asthma. However, the availability of large multigenerational pedigrees remains a major hurdle, especially in complex diseases such as asthma or atopy. Heterogeneity among different populations for genetic factors contributing to complex traits such as asthma or atopy also complicates linkage analysis.

The linkage and haplotype analyses using a panel of SNPs in three adjacent chemokine receptor genes as markers in this study suggested that there was an association between chromosome 3 and asthma. However, a larger data set would be required to confirm the suggestions from these haplotype analyses due to the low number of individuals in some of haplotype fields.
